# A Screen for Epigenetically Silenced microRNA Genes in Gastrointestinal Stromal Tumors

**DOI:** 10.1371/journal.pone.0133754

**Published:** 2015-07-27

**Authors:** Mai Isosaka, Takeshi Niinuma, Masanori Nojima, Masahiro Kai, Eiichiro Yamamoto, Reo Maruyama, Takayuki Nobuoka, Toshirou Nishida, Tatsuo Kanda, Takahiro Taguchi, Tadashi Hasegawa, Takashi Tokino, Koichi Hirata, Hiromu Suzuki, Yasuhisa Shinomura

**Affiliations:** 1 Department of Gastroenterology, Rheumatology and Clinical Immunology, Sapporo Medical University School of Medicine, Sapporo, Japan; 2 Department of Molecular Biology, Sapporo Medical University, Sapporo, Japan; 3 Center for Translational Research, The Institute of Medical Science, The University of Tokyo, Tokyo, Japan; 4 Department of Surgery, Surgical Oncology and Science, Sapporo Medical University School of Medicine, Sapporo, Japan; 5 National Cancer Center Hospital East, Kashiwa, Japan; 6 Department of Surgery, Sanjo General Hospital, Sanjo City, Niigata, Japan; 7 Division of Human Health and Medical Science, Graduate School of Kuroshio Science, Kochi University, Nankoku, Japan; 8 Department of Surgical Pathology, Sapporo Medical University School of Medicine, Sapporo, Japan; 9 Medical Genome Science, Research Institute for Frontier Medicine, Sapporo Medical University School of Medicine, Sapporo, Japan; Oklahoma State University, UNITED STATES

## Abstract

**Background:**

Dysregulation of microRNA (miRNA) has been implicated in gastrointestinal stromal tumors (GISTs) but the mechanism is not fully understood. In this study, we aimed to explore the involvement of epigenetic alteration of miRNA genes in GISTs.

**Methods:**

GIST-T1 cells were treated with 5-aza-2’-deoxycytidine (5-aza-dC) and 4-phenylbutyric acid (PBA), after which miRNA expression profiles were analyzed using TaqMan miRNA arrays. DNA methylation was then analyzed using bisulfite pyrosequencing. The functions of miRNAs were examined using MTT assays, wound-healing assays, Boyden chamber assays and Matrigel invasion assays. Gene expression microarrays were analyzed to assess effect of ectopic miRNA expression in GIST-T1 cells.

**Results:**

Of the 754 miRNAs analyzed, 61 were significantly upregulated in GIST-T1 cells treated with 5-aza-dC plus PBA. Among those, 21 miRNA genes were associated with an upstream CpG island (CGI), and the CGIs of miR-34a and miR-335 were frequently methylated in GIST-T1 cells and primary GIST specimens. Transfection of miR-34a or miR-335 mimic molecules into GIST-T1 cells suppressed cell proliferation, and miR-34a also inhibited migration and invasion by GIST-T1 cells. Moreover, miR-34a downregulated a number of predicted target genes, including *PDGFRA*. RNA interference-mediated knockdown of *PDGFRA* in GIST-T1 cells suppressed cell proliferation, suggesting the tumor suppressive effect of *miR-34a* is mediated, at least in part, through targeting *PDGFRA*.

**Conclusions:**

Our results suggest that miR-34a and miR-335 are candidate tumor suppressive miRNAs in GISTs, and that they are frequent targets of epigenetic silencing in GISTs.

## Introduction

Gastrointestinal stromal tumors (GISTs) are the most common mesenchymal tumors of the gastrointestinal tract [1]. GISTs occur predominantly in the stomach (50–60%) and small intestine (30–35%), and are thought to originate from interstitial cells of Cajal (ICC) or their precursor cells. More than 80% of GISTs have a gain of function mutation within *KIT*, which results in the constitutive activation of the c-KIT receptor. Alternatively, approximately one-third of GISTs with no *KIT* mutations carry mutations in *PDGFRA*, which encodes platelet-derived growth factor receptor α. In addition to *KIT* and *PDGFRA* mutations, a majority of GISTs acquire other genetic and epigenetic abnormalities during their malignant progression. For instance, earlier cytogenetic, fluorescence in situ hybridization (FISH) and comparative genomic hybridization (CGH) studies revealed frequent losses at 14q and 22q [2]. Moreover, recent array CGH analyses identified a number of chromosomal imbalances that could be relevant to the pathogenesis of GISTs [2, 3]. In addition to genetic alterations, aberrant DNA methylation has also been implicated in the development of GISTs. We previously showed that hypomethylation of repetitive sequences, including LINE-1, correlates with increased chromosomal aberration and GIST malignancy [4], and a recent genome-wide DNA methylation analysis revealed that hypermethylation of three genes (*REC8*, *PAX3* and *p16*) is strongly associated with aggressive clinical behavior and an unfavorable prognosis [5].

MicroRNAs (miRNAs) are a group of small noncoding RNAs that negatively regulate the translation and stability of partially complementary target messenger RNAs [6]. miRNAs are highly conserved among species and play critical roles in a variety of biological processes, including development, differentiation, cell proliferation and apoptosis. Consistent with their role in these processes, a number of studies have shown widespread alterations in the expression patterns of miRNAs in various malignancies, including GISTs [7, 8]. For instance, the patterns of miRNA expression in GISTs reflect the status of 14q loss, tumor locations and risk grades [9, 10]. We recently reported that elevated miR-196a expression is tightly associated with the malignant characteristics of GISTs [11], while other groups have reported downregulated expression of the putative tumor suppressors miR-137 and miR-218 in GISTs [12, 13]. Although the mechanisms underlying miRNA dysregulation in cancer are not yet fully understood, recent studies have shown that the silencing of several miRNAs is tightly linked to epigenetic mechanisms, including histone modification and DNA methylation. Treatment with histone deacetylase (HDAC) and DNA methyltransferase (DNMT) inhibitors restored expression of various miRNAs in cancer cells, and the list of miRNA genes methylated in cancer is rapidly growing [14]. When we screened for epigenetically silenced miRNA genes in colorectal (CRC), gastric and bladder cancers, we found a number of miRNA genes silenced in association with methylation of CpG islands (CGIs) in their promoter regions [15–18]. However, the involvement of epigenetic alterations in the dysregulation of miRNAs in GIST has not been reported up to now.

In the present study, we aimed to identify miRNAs associated with the pathogenesis of GISTs. To that end, we searched for miRNA genes epigenetically silenced in GIST cells by screening for miRNAs whose expression was upregulated by DNA demethylation and HDAC inhibition. We found that miR-34a and miR-335 are frequent targets of epigenetic silencing in GISTs, and that they may act as suppressors of GIST development.

## Material and Methods

### Cell line and tissue samples

GIST-T1 cells have been described elsewhere [19]. The cells were treated first for 72 h with a 2 μM concentration of the DNMT inhibitor 5-aza-2’-deoxycytidine (5-aza-dC; Sigma-Aldrich), and then for 48 h with a 3 mM concentration of the HDAC inhibitor 4-phenylbutyric acid (PBA; Sigma-Aldrich), replacing the drug and medium every 24 h. Thirty-nine fresh frozen GIST specimens were obtained from Sapporo Medical University Hospital and Osaka University Hospital, and formalin-fixed, paraffin-embedded tissue sections from 98 GIST specimens were obtained from Niigata University Hospital, as described previously [11]. Informed consent was obtained from all patients before collection of the specimens, and this study was approved by the institutional review board. Risk grades were assigned based on tumor size and mitotic activity using the risk classification system proposed by Fletcher et al. [20]. Total RNA was extracted using TRIZOL reagent (Invitrogen) or RNeasy Mini Kits (Qiagen). Genomic DNA was extracted using the standard phenol-chloroform procedure.

### miRNA expression analysis

Expression of a set of 754 miRNAs was examined using a TaqMan microRNA Array v3.0 (Applied Biosystems). The PCR was run in a 7900HT Fast Real-Time PCR System (Applied Biosystems), and SDS 2.2.2 software (Applied Biosystems) was used for comparative delta Ct analysis. U6 snRNA (Applied Biosystems) was used as an endogenous control.

### DNA methylation analysis

Genomic DNA (1 μg) was modified with sodium bisulfite using an EpiTect Bisulfite Kit (Qiagen), after which methylation analysis was carried out as described previously [17]. For bisulfite pyrosequencing, the biotinylated PCR product was purified, made single-stranded and used as the template in a pyrosequencing reaction run according to the manufacturer’s instructions. The pyrosequencing reaction was carried out using a PSQ96 system with a PyroGold reagent kit (Qiagen), and the results were analyzed using Q-CpG software (Qiagen). Sequence information for primers is shown in S1 Table.

### Transfection of miRNA mimics and siRNA

GIST-T1 cells (3 x 10^6^) were transfected with 100 pmol of mirVana miRNA mimics (Ambion) or mirVana miRNA mimic Negative Control #1 (Ambion) using a Cell Line Nucleofector kit L (Lonza) and a Nucleofector I electroporation device (Lonza) according to manufacturer’s instructions. For RNA interference-mediated knockdown of *PDGFRA*, cells were transfected with 100 pmol of a Silencer Select siRNA targeting *PDGFRA* (Applied Biosystems) or a Silencer Select Negative Control (Applied Biosystems) using a Cell Line Nucleofector kit L (Lonza).

### Cell viability assay

GIST-T1 cells were transfected with miRNA mimics or siRNA as described above, and seeded into 96-well plate to a density of 1 x 10^5^ cells per well. After incubation for 72 h, cell viability was examined using a Cell Counting kit-8 (Dojindo) according to the manufacturer’s instructions.

### Wound healing assay

GIST-T1 cells were transfected with miRNA mimics or a negative control as described above. Cells were then seeded onto 35-mm dishes containing a Culture-Insert (Ibidi). The insert was removed 24 h after transfection, leaving a 0.5 mm cell free wound field. Photographs of cells invading the wound area were taken at the indicated times, and wound areas were measured using the ImageJ software (NIH).

### Cell invasion and migration assays

For Matrigel invasion assays, GIST-T1 cells were transfected with miRNA mimics or a negative control as described above, after which 5 x 10^4^ transfectant cells were suspended in 500 μL of serum-free Dulbecco’s Modified Eagle medium (DMEM) (Sigma-Aldrich) and added to the tops of BD BioCoat Matrigel Invasion Chambers (BD Biosciences) prehydrated with phosphate-buffered saline (PBS), and 700 μL of DMEM supplemented with 10% fetal bovine serum (FBS) were added to the lower wells of the plate. For migration assays, a control insert (BD Biosciences) was used instead of a Matrigel Invasion Chamber. After incubation for 24 h, invading or migrating cells were stained and counted in five randomly selected microscope fields per membrane.

### Gene expression microarray analysis

GIST-T1 cells were transfected with miRNA mimics or a negative control as described above, and total RNA was extracted 48 h later. One-color microarray-based gene expression analysis was then carried out according to manufacturer’s instructions (Agilent Technologies). Briefly, 100 ng of total RNA were amplified and labeled using a Low-input Quick Amp Labelling kit One-color (Agilent Technologies), after which the synthesized cRNA was hybridized to a SurePrint G3 Human GE microarray v2 (G4851; Agilent Technologies). The microarray data were analyzed using GeneSpring GX version 13 (Agilent Technologies). The genes targeted by the miRNAs were predicted using the TargetScan system integrated into the GeneSpring GX software package. The Gene Expression Omnibus accession number for the microarray data is GSE68743.

### Luciferase reporter assay

Oligonucleotides containing the two putative miR-34a target sites in the 3’ untranslated region (UTR) of *PDGFRA* or mutant target sites were annealed, digested using *Spe*I and *Hin*dIII and cloned into pMIR-REPORT (Ambion) according to the manufacturer’s instructions. The sequences of the oligonucleotides are listed in S1 Table. GIST-T1 cells (1×10^5^ cells/well in 24-well plates) were transfected with 100 ng of one of the reporter plasmids, 1 ng of pRL-CMV (Promega) and 15 pmol of a miRNA mimic or a negative control using Lipofectamine 3000 (Invitrogen). Luciferase activities were then measured 48 h after transfection using a Dual-Luciferase Reporter Assay System (Promega).

### Quantitative RT-PCR

Single-stranded cDNA was prepared using SuperScript III reverse transcriptase (Invitrogen). Quantitative reverse transcription PCR (RT-PCR) of *PDGFRA* was carried out using a TaqMan Gene Expression Assay (Assay ID, Hs00998018_m1; Applied Biosystems) and a 7500 Fast Real-Time PCR System (Applied Biosystems). *GAPDH* (Assay ID, Hs02758991_g1; Applied Biosystems) served as an endogenous control.

### Statistical analysis

Comparisons of continuous variables were made using t tests or one-way ANOVA with post-hoc multiple comparisons (Tukey HSD test). *P* values of <0.05 (two-sided) were considered significant. All data were analyzed using SPSS Statistics 20 (IBM Corporation) or GraphPad Prism version 5.02 (GraphPad Software).

## Results

### Identification of miRNA genes epigenetically silenced in GIST-T1 cells

**Fig 1:**
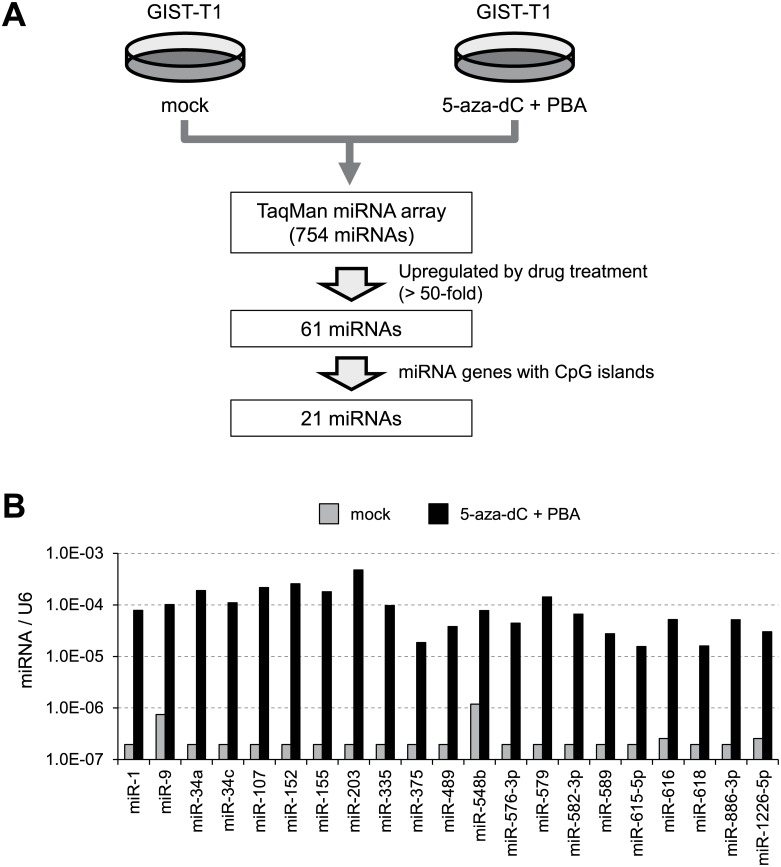
Identification of epigenetically silenced miRNAs in GIST-T1 cells. (A) Workflow of the screen to identify epigenetically silenced miRNAs. (B) Summarized TaqMan array results for 22 candidate miRNAs in GIST-T1 cells treated with or without 5-aza-dC plus PBA. Expression levels were normalized to that of U6 snRNA expression.

**Fig 2:**
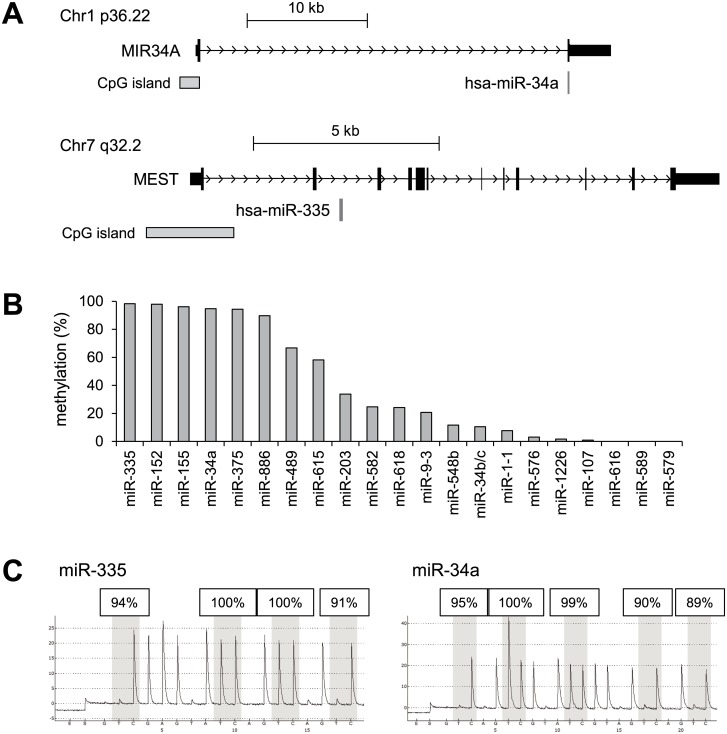
Analysis of CGI methylation at predicted transcription start sites of miRNA genes. (A) Representative examples of miRNA gene structures. Host genes encoding miR-34a and miR-335 are shown. CGIs and pre-miRNA regions are shown below. (B) Levels of CGI methylation in the selected miRNA genes were analyzed using bisulfite pyrosequencing in GIST-T1 cells. (C) Results of bisulfite pyrosequencing of miR-335 and miR-34a in GIST-T1 cells.

To identify miRNA genes epigenetically silenced in GIST, we assessed miRNA expression profiles in GIST-T1 cells treated with or without the DNMT inhibitor 5-aza-dC plus the HDAC inhibitor PBA (Fig 1A). Of the 754 miRNAs analyzed, 61 were expressed at low levels in GIST-T1 cells and were significantly upregulated (>50-fold) by the drug treatment (S2 Table). We excluded miRNA genes located on X chromosomes from further analysis, as well as miRNA genes in the miRNA cluster on chromosome 19, which are placenta-specific and are epigenetically silenced in normal adult tissues. Among the upregulated miRNAs, we selected the 21 in which the predicted transcription start sites were associated with a CGI (Figs 1B and 2A). We next used bisulfite pyrosequencing to analyze the methylation status of the CGIs in the selected miRNA genes in GIST-T1 cells (Fig 2B and 2C). As summarized in Fig 2B, 6 miRNA genes (miR-335, miR-152, miR155, miR-34a, miR-375 and miR-886) were almost fully methylated in GIST-T1 cells, and another 6 genes (miR-489, miR-615, miR-203, miR-582, miR-618 and miR-9-3) were partially methylated to some degree. The CGIs of the remaining 9 genes were methylated at low levels (<15%) or were completely unmethylated, indicating that CGI methylation was likely not be the major mechanism underlying the silencing of these genes.

### Methylation analysis of miRNA genes in primary GIST tumors

**Fig 3:**
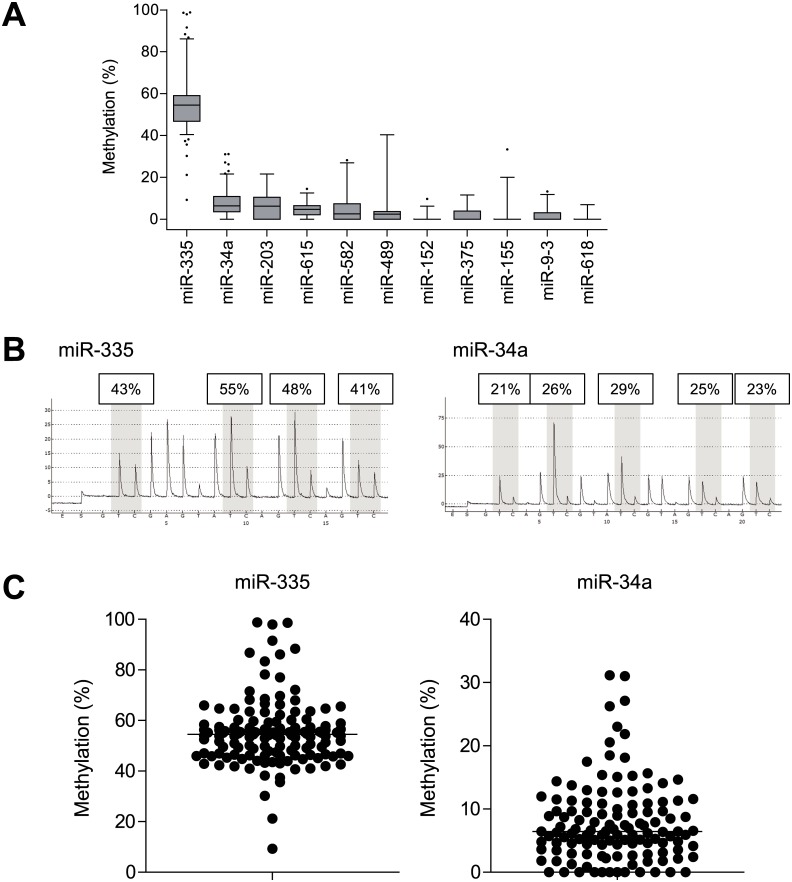
Analysis of miRNA gene methylation in primary GIST specimens. (A) Summarized results showing the methylation levels of selected miRNAs in primary tumors analyzed using bisulfite pyrosequencing. (B) Representative results of bisulfite pyrosequencing of miR-335 and miR-34a in primary tumors. (C) Summarized results showing bisulfite pyrosequencing of miR-335 and miR-34a in primary GIST specimens.

We next analyzed the methylation of miRNA genes in primary GIST specimens. Of the 12 miRNA genes methylated in GIST-T1 cells, a precursor of miR-886 (pre-miR-886) was recently reported to be a novel noncoding RNA [21] and was deleted from the miRNA database (http://www.mirbase.org/). We therefore excluded miR-886 from further analysis, and carried out bisulfite pyrosequencing analysis of the remaining 11 miRNA genes in a series of primary GIST specimens (Fig 3A and 3B, Table 1). As summarized in Fig 3A, miR-335 was methylated to the greatest degree among the 11 genes, and the majority of the GIST specimens exhibited significantly elevated levels of miR-335 methylation (Fig 3C). Subsequent bisulfite sequencing in selected samples confirmed that the CGI region of miR-335 was densely methylated in both GIST-T1 cells and primary tumors (S1 File). We also noted that miR-34a, which is known to be tumor suppressive and is downregulated in various types of malignancies, was also methylated (>10%) in 33 of 123 GIST specimens (26.8%) (Fig 3C). In addition, when we analyzed the relationship between miRNA gene methylation and the clinicopathological features of GIST patients, methylation of neither gene correlated with age, gender, risk grade, metastasis, tumor size or mitotic counts (Table 1). Levels of miR-335 methylation were lower in tumors in stomach and small intestine than in the esophagus or colon, but due to the limited numbers of samples in the latter groups, significant association was not observed by the post-hoc pair-wise test (Table 1). miR-335 methylation was also lower in low-risk and very low-risk tumors, although not to a statistically significant degree (Table 1).

**Table 1:** Correlation between methylation of miRNA genes and the clinicopathological features of GIST patients.

		miR-34a methylation (%)				miR-335 methylation (%)			
		N	Mean	SD	*P*	N	Mean	SD	*P*
Age	≤65	60	7.5	6.0		66	56.8	14.3	
	>65	63	8.2	6.8	0.579*	68	53.4	12.0	0.146*
Gender	Male	58	7.7	6.8		62	55.6	15.1	
	Female	64	8.0	5.9	0.805*	72	54.6	11.6	0.677*
Tumor location	Esophagus	4	1.8	3.7		4	71.4	19.1	
	Stomach	98	8.1	6.4		108	54.6	13.7	
	Intestine	17	9.0	5.9		18	53.1	7.1	
	Colon	2	1.2	1.2	0.083#	2	66.6	4.4	0.045# ¶
Risk grade	High risk	37	8.1	8.0		43	57.5	13.3	
	Intermediate risk	29	6.7	5.6		33	57.4	14.7	
	Low or very low risk	54	8.3	5.6	0.518#	55	52.4	11.9	0.094#
Metastasis	+	22	8.1	7.8		24	57.4	14.8	
	-	100	7.8	6.1	0.824*	102	54.4	13.3	0.328*
Tumor size (cm)	≤5.0	43	7.7	5.3		50	54.7	13.0	
	>5.0	75	8.4	8.1	0.553*	79	88.8	14.4	0.655*
Mitotic count (/50 HPF)	≤5	97	7.6	6.1		99	54.5	12.6	
	>5	15	9.8	9.0	0.231*	21	59.9	18.2	0.099*

*P value was determined using Student’s t test.

^#^ *P* value was determined using ANOVA.

^¶^Overall testing by ANOVA was statistically significant, but no significant difference was observed by the post-hoc pair-wise Tukey HSD comparison.

### Functional analysis of miR-34a and miR-335

**Fig 4:**
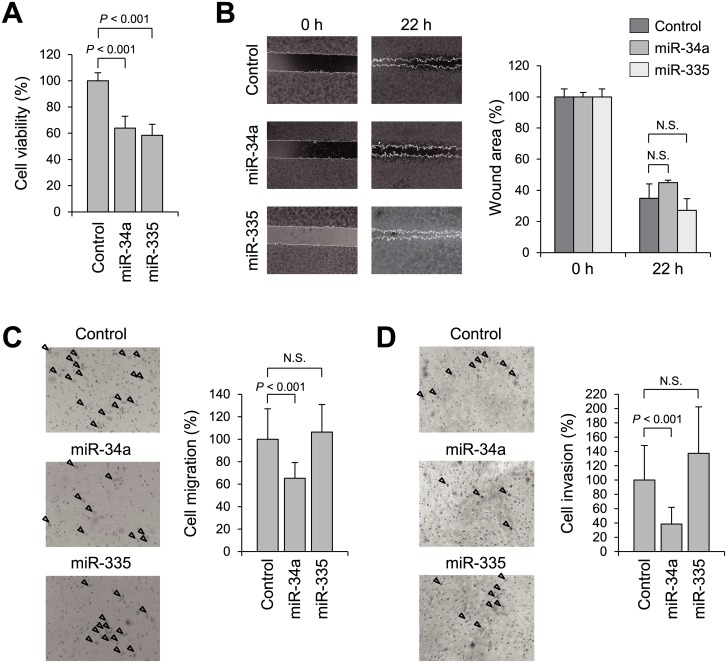
Functional analysis of miR-34a and miR-335. (A) Cell viability assays using GIST-T1 cells transfected with miR-34a or miR-335 mimics or a negative control. Cell viabilities were determined 72 h after transfection. Shown are the means of 8 replications; error bars represent standard deviations; *P* values were determined using Student’s t test. (B) Wound healing assay using GIST-T1 cells transfected with a miRNA mimic or a negative control. Shown on the right are the means of 4 replications; error bars represent standard deviations; *P* values were determined using Student’s t test. (C,D) Cell migration (C) and Matrigel invasion (D) assays using GIST-T1 cells transfected with a miRNA mimic or a negative control. Arrowheads indicate migrating or invading cells. Shown on the right are the means of 5 random microscopic fields per membrane; error bars represent standard deviations; *P* values were determined using Student’s t test.

To investigate whether miR-34a and/or miR-335 act as tumor suppressors in GIST, we transfected GIST-T1 cells with miRNA mimic molecules or a negative control, and then carried out cell viability, migration and invasion assays. We found that 72 h after transfection, ectopic expression of miR-34a and miR-335 suppressed GIST-T1 cell growth (Fig 4A). In wound healing assays, GIST-T1 cells expressing ectopic miR-34a tended to migrate toward the wound more slowly than control cells, though the effect was not statistically significant (Fig 4B). Boyden chamber assays showed that miR-34a suppressed migration and Matrigel invasion by GIST-T1 cells (Fig 4C and 4D). By contrast, miR-335 did not suppress cell migration or invasion (Fig 4B and 4C).

**Fig 5:**
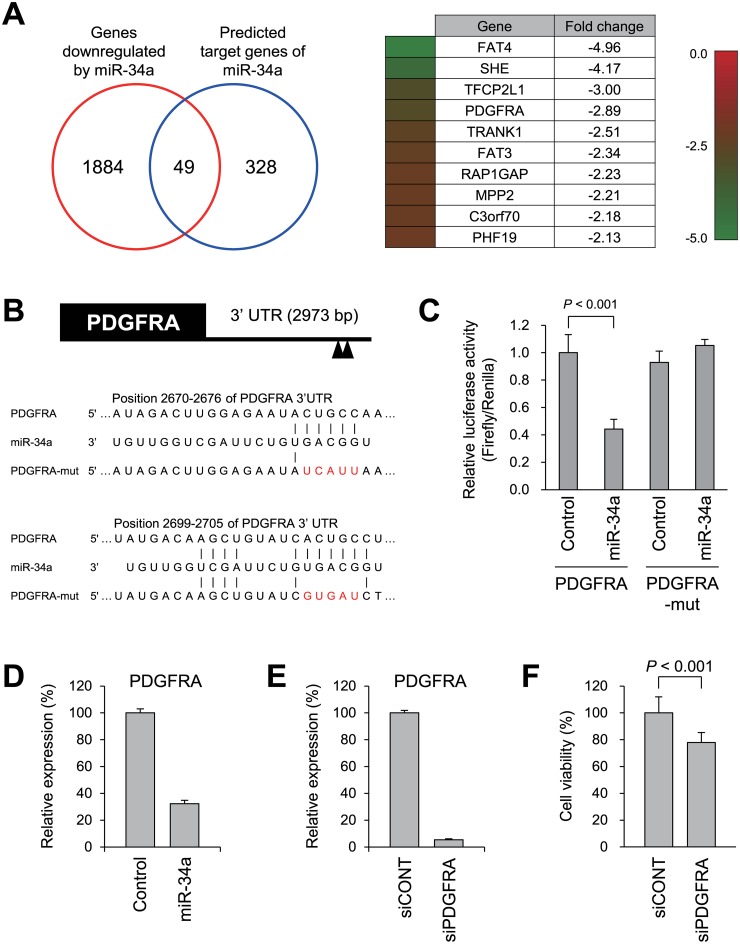
Downregulation of predicted miR-34a target genes in GIST-T1 cells. (A) Venn diagram for genes downregulated by ectopic miR-34a expression in GIST-T1 cells (>1.5-fold) and predicted miR-34a target genes. Of the 49 downregulated target genes, the top 10 genes are listed on the right. Expression levels and fold-changes are also indicated. (B) Putative miR-34a binding sites in the 3’ untranslated region (UTR) of *PDGFRA*. Mutant binding sites used for the luciferase assay are shown in red. (C) Reporter assay results using a luciferase vector containing the wild-type *PDGFRA* 3’ UTR (PDGFRA) or the mutant 3’ UTR (PDGFRA-mut) in GIST-T1 cells cotransfected a miR-34a mimic or a negative control. Shown are means of 4 replications; error bars represent standard deviations; the *P* value was determined using Student’s t test. (D) Quantitative RT-PCR of *PDGFRA* in GIST-T1 cells transfected with a miR-34a mimic or a negative control. (E) Quantitative RT-PCR of *PDGFRA* in GIST-T1 cells transfected with a siRNA targeting *PDGFRA* (siPDGFRA) or a control siRNA (siCONT). (F) Cell viability assays using GIST-T1 cells transfected with siCONT or siPDGFRA. Cell viabilities were determined 72 h after transfection. Shown are means of 8 replications; error bars represent standard deviations; the *P* value was determined using Student’s t test.

To further clarify the effect of these miRNAs, we also carried out a gene expression microarray analysis of GIST-T1 cells transfected with miRNA mimic molecules or a negative control. We found that 2,621 probe sets representing 1,933 unique genes were downregulated (>1.5-fold) by ectopic miR-34a expression. Gene Ontology (GO) analysis revealed that “anchored component of membrane,” “epidermal cell differentiation” and “multicellular organismal development” genes were enriched among the downregulated genes (S3 Table). Microarray analysis also revealed that that 49 predicted miR-34a target genes were downregulated by a miR-34a miRNA mimic in GIST-T1 cells (Fig 5A, S1 File). Among those, we focused on *PDGFRA*, which has been strongly implicated in the pathogenesis of GIST [22]. *PDGFRA* mRNA contains putative miR-34a binding sites in its 3’ UTR [23, 24] (Fig 5B). Reporter assays using a luciferase vector containing wild-type miR-34a binding sites revealed that cotransfection of a miR-34a mimic markedly downregulated luciferase activity in GIST-T1 cells (Fig 5C). Such downregulation was not observed when cells were transfected with a luciferase vector containing mutant miR-34a binding sites (Fig 5B and 5C). Suppression of *PDGFRA* expression by miR-34a was further confirmed by quantitative RT-PCR using GIST-T1 cells (Fig 5D). In addition, *PDGFRA* knockdown using a specific siRNA suppressed the viability of GIST-T1 cells, suggesting the tumor suppressive effect of miR-34a may be mediated at least in part through targeting *PDGFRA* (Fig 5E and 5F).

We also performed a gene expression microarray analysis using GIST-T1 cells transfected with a miR-335 mimic, and found that 1,095 probes representing 853 unique genes were downregulated (>1.5-fold). Gene ontology analysis showed that “D-aspartate transport,” “neuronal action potential” and “epidermis development” genes were enriched among the downregulated genes (S4 Table). Microarray analysis also revealed that 16 predicted target genes, including *EIF5A2*, *ZMPSTE24* and *MAT2B*, were downregulated by miR-335 in GIST-T1 cells (S1 File).

## Discussion

In this study, we tried to identify miRNA genes that are potentially inactivated through an epigenetic mechanism in GIST cells. Despite applying a stringent criterion (>50-fold induction by 5-aza-dC plus PBA) in the first step of our screening, we found 61 miRNAs to be upregulated by the drug treatment in GIST-T1 cells, indicating that a large number of miRNAs may be epigenetically regulated in GIST cells. We then selected 21 miRNA genes in which the predicted promoter regions contains a CGI, and found that 6 of those genes were methylated at high levels in GIST-T1 cells. In an earlier integrative analysis of miRNA expression profiles and genome-wide chromatin signatures of miRNA genes in CRC cells, we observed a similar discrepancy between the number of miRNAs upregulated in tumor cells by epigenetic drug treatment and the number of genes methylated [17]. Although more than 100 miRNAs were upregulated by DNA demethylating treatment in CRC cells, we ultimately identified CGI methylation of only 22 primary miRNA genes. One possible explanation for this discrepancy is that the transcription start sites of miRNA genes, especially those in intergenic regions, are not fully identified. A second possibility is that mechanisms other than DNA methylation, such as repressive histone modification, may be associated with the miRNA gene silencing. In addition, some miRNAs may be upregulated due to indirect effects of the epigenetic drug treatment.

We found that the promoter CGI of miR-335 was frequently methylated in GISTs. Several studies have implicated miR-335 in human malignancies. For example, the tumor suppressive functions of miR-335 have been reported in breast, lung, pancreatic, gastric and ovarian cancers and neuroblastoma [25–29]. miR-335 suppresses metastasis and/or invasion in gastric and ovarian cancer by targeting Bcl-w [27, 28], and it inhibits small cell lung cancer metastasis by targeting IGF-IR and RANKL [26]. miR-335 also suppresses neuroblastoma cell invasiveness by targeting multiple genes encoding mediators in the TGF-pathway [29], and it is involved in the regulation of *BRCA1* in breast cancer [30] and is silenced through genetic and epigenetic mechanisms in metastatic breast cancer cells [25]. In addition, miR-335 and its host gene, *MEST*, are silenced in association with CGI methylation in hepatocellular carcinoma (HCC) [31], and replacement of miR-335 exerts tumor suppressive effects in lung, pancreatic and breast cancer cells, which is suggestive of the therapeutic potential of miR-335 [32–34]. In contrast to the observations summarized above, several studies suggest miR-335 exerts oncogenic effects. Strong expression of miR-335 is reportedly associated with a poor prognosis in gastric cancer and glioma [35, 36], and the tumor promoting actions of miR-335 have been demonstrated in astrocytoma cells, suggesting that it could be a potential target for the treatment of astrocytoma [37]. These results indicate that miR-335 may play opposite roles during tumorigenesis in different organs.

In the present study, although miR-335 did not inhibit migration or invasion by GIST-T1 cells, it did suppress the viability of GIST cells. Gene expression microarray analysis revealed that 16 predicted target genes were downregulated by ectopic miR-335 expression. Among them, *EIF5A2* (eukaryotic initiation factor 5A2) is a candidate oncogene isolated from the amplified region at 3q in ovarian cancer [38]. *EIF5A* is also reportedly overexpressed in lung cancer, HCC, esophageal cancer and CRC, and it is associated with cancer cell aggressiveness and metastasis [39–42]. MAT2B is a regulatory subunit of the cellular enzyme methionine adenosyltransferase (MAT), which catalyzes the synthesis of S-adenosylmethionine and is involved in the growth of HCC and CRC [43]. ZMPSTE24 is a metalloproteinase mutated in human progeria and is involved in nuclear prelamin A maturation [44]. A recent study showed that silencing of *ZMPSTE24* reduces cancer cell invasion, suggesting that it could potentially serve as a new therapeutic target [45]. The results from these reports together with our present findings are indicative of the potential tumor suppressive roles of miR-335 in GISTs.

In this study, we also found that miR-34a CGI is methylated in cultured and primary GIST cells. Although the degree and frequency of miR-34a methylation in clinical GISTs were much lower than those of miR-335 methylation, miR-34a is located at 1p36, which is frequently deleted in GISTs [2]. The miR-34 family has been strongly implicated in tumorigenesis. All three miR-34 family members (miR-34a, -34b and -34c) are directly regulated by p53, and ectopic expression of miR-34s induces cell cycle arrest and/or apoptosis in human cancer cells [46–49]. The tumor suppressive activity of miR-34a has been reported in CRC, HCC, pancreatic cancer and glioblastoma [50–53]. We also found that ectopic expression of miR-34a in GIST cells suppresses cell proliferation, migration and invasion. Inactivation of miR-34a due to CGI methylation has been observed in malignancies of the colon, pancreas, breast, ovary, urinary tract and kidney, and in hematological malignancies [54, 55]. Taken together with these reports, our findings suggest that miR-34a may act as a tumor suppressor in GIST, and epigenetic silencing of miR-34a may contribute to GIST development. Given its tumor suppressive function, restoration of miR-34a expression would seem to be a potentially therapeutic strategy. In mouse xenograft models of lung cancer, systemic delivery of a miR-34a mimic using a lipid-based delivery vehicle inhibited tumor growth [56]. miR-34a also reportedly inhibits prostate cancer stem cells, and the therapeutic efficacy of its systemic delivery has been confirmed in a mouse xenograft model [57]. Our results thus suggest miRNA replacement therapy may be an effective therapeutic strategy for the treatment of GISTs.

miR-34a regulates multiple cellular processes by targeting genes involved in cell cycle, differentiation, apoptosis and growth signaling [58]. Using gene expression microarray analysis, we found that 49 predicted miR-34a target genes were downregulated by ectopic miR-34a expression in GIST-T1 cells. Among them, *PDGFRA* was recently reported to be a direct target of miR-34a in malignant glioma, lung cancer and gastric cancer [23, 24, 59]. It is well documented that gain-of-function mutations in *KIT* or *PDGFRA* play essential roles in the development of GISTs [1]. In that context, imatinib (formerly STI571), which was developed as a tyrosine kinase inhibitor and was found to inhibit KIT and PDGFRA, is currently being used for the treatment of metastatic GISTs [1]. Our data suggest that loss of miR-34a may be an alternative event leading to the activation of *PDGFRA* in GIST cells, and that replacement of miR-34a could potentially exert a therapeutic effect through downregulation of *PDGFRA*. We observed that *PDGFRA* knockdown in GIST-T1 cells suppressed cell viability, though the effect was relatively limited, as compared to that of miR-34a. GIST-T1 cells harbor a gain of function mutation in exon 11 of the *KIT* gene [60], and the effect of *PDGFRA* downregulation may be limited in GIST cells with the *KIT* mutation. Further studies will be necessary to clarify the functional role and clinical significance of miR-34a inactivation in GISTs.

## Conclusions

We have shown that miR-335 and miR-34a are targets of epigenetic silencing in GISTs. This is the first report of epigenetic silencing of miRNA genes in this disease. Although their specific functions in the development of GISTs are not yet fully understood, restoration of miR-335 and miR-34a may be an effective anticancer therapy. Further study of miRNA dysregulation and its functional significance in GISTs may provide new strategies for the treatment of GIST patients.

## Supporting Information

S1 File Fig A in S1 File. Representative bisulfite sequencing results for the CGI in the miR-335 gene promoter in GIST-T1 cells and primary GIST specimens. Open and filled circles represent unmethylated and methylated CpG sites, respectively. Fig B in S1 File. Venn diagram for genes downregulated by ectopic miR-34a expression in GIST-T1 cells (>1.5-fold) and predicted miR-34a target genes. Downregulated target genes are listed on the right. Fig C in S1 File. Venn diagram for genes downregulated by ectopic miR-335 expression in GIST-T1 cells (>1.5-fold) and predicted miR-335 target genes. Downregulated target genes are listed on the right.
(DOC)

S1 Table Primer sequences and PCR product sizes used in this study. (XLSX)

S2 Table miRNAs upregulated by 5-aza-dC plus PBA in GIST-T1 cells. (XLSX)

S3 Table GO analysis of downregulated genes in GIST-T1 with ectopic miR-34a expression. (XLSX)

S4 Table GO analysis of downregulated genes in GIST-T1 with ectopic miR-335 expression. (XLSX)
