# Supplementary material for: A Screen for Epigenetically Silenced microRNA Genes in Gastrointestinal Stromal Tumors
Source: PLoS One. 2015 Jul 27;10(7):e0133754. doi: 10.1371/journal.pone.0133754 (PMC4516245; doi:10.1371/journal.pone.0133754)
Supplement: S1 File — Fig A in S1 File. Representative bisulfite sequencing results for the CGI in the miR-335 gene promoter in GIST-T1 cells and primary GIST specimens. Open and filled circles represent unmethylated and methylated CpG sites, respectively. Fig B in S1 File. Venn diagram for genes downregulated by ectopic miR-34a expression in GIST-T1 cells (>1.5-fold) and predicted miR-34a target genes. Downregulated target genes are listed on the right. Fig C in S1 File. Venn diagram for genes downregulated by ectopic miR-335 expression in GIST-T1 cells (>1.5-fold) and predicted miR-335 target genes. Downregulated target genes are listed on the right. (DOC) [file pone.0133754.s001.doc]

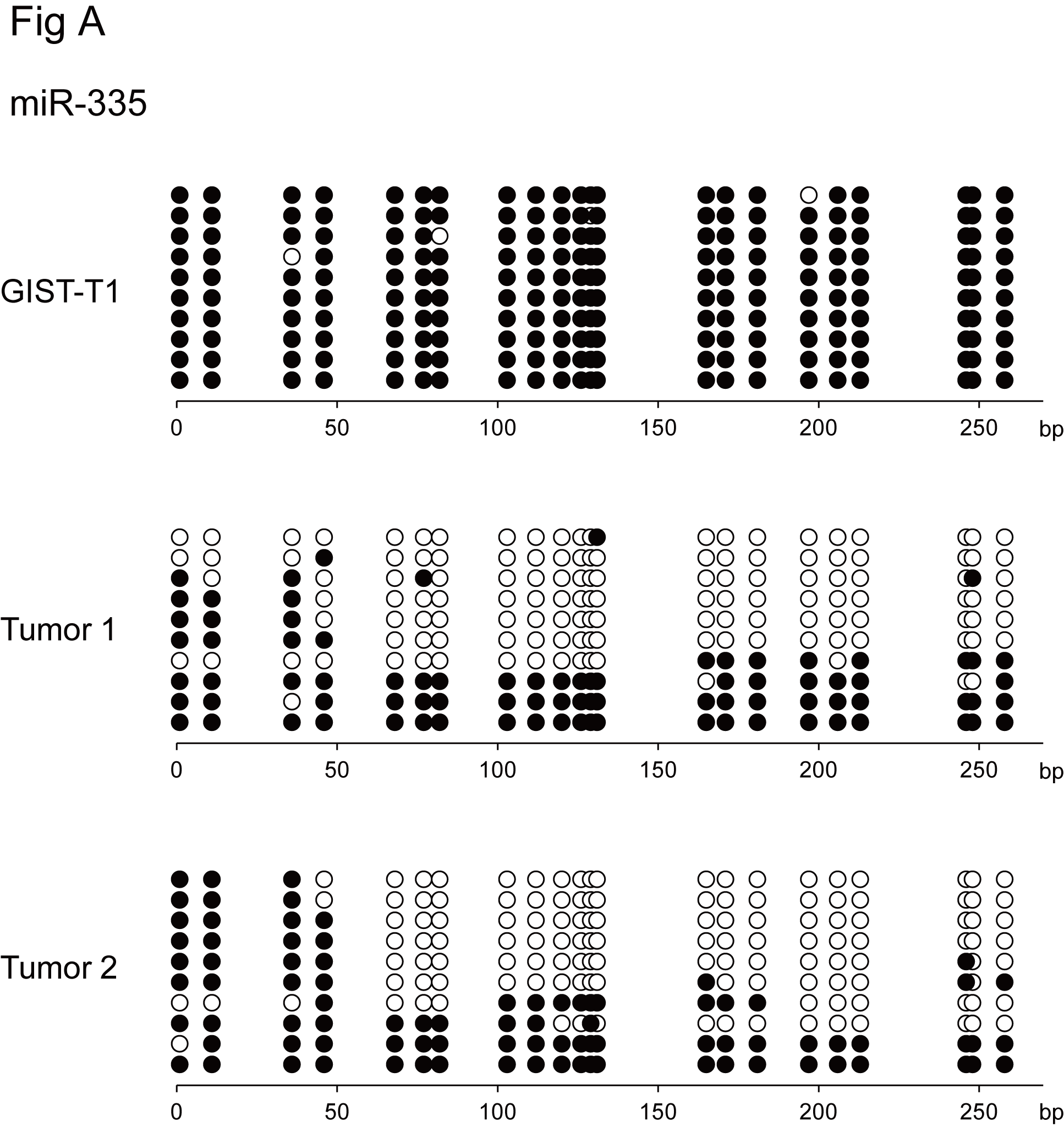


**Fig A**

Representative bisulfite sequencing results for the CGI in the miR-335 gene promoter in GIST-T1 cells and primary GIST specimens. Open and filled circles represent unmethylated and methylated CpG sites, respectively.


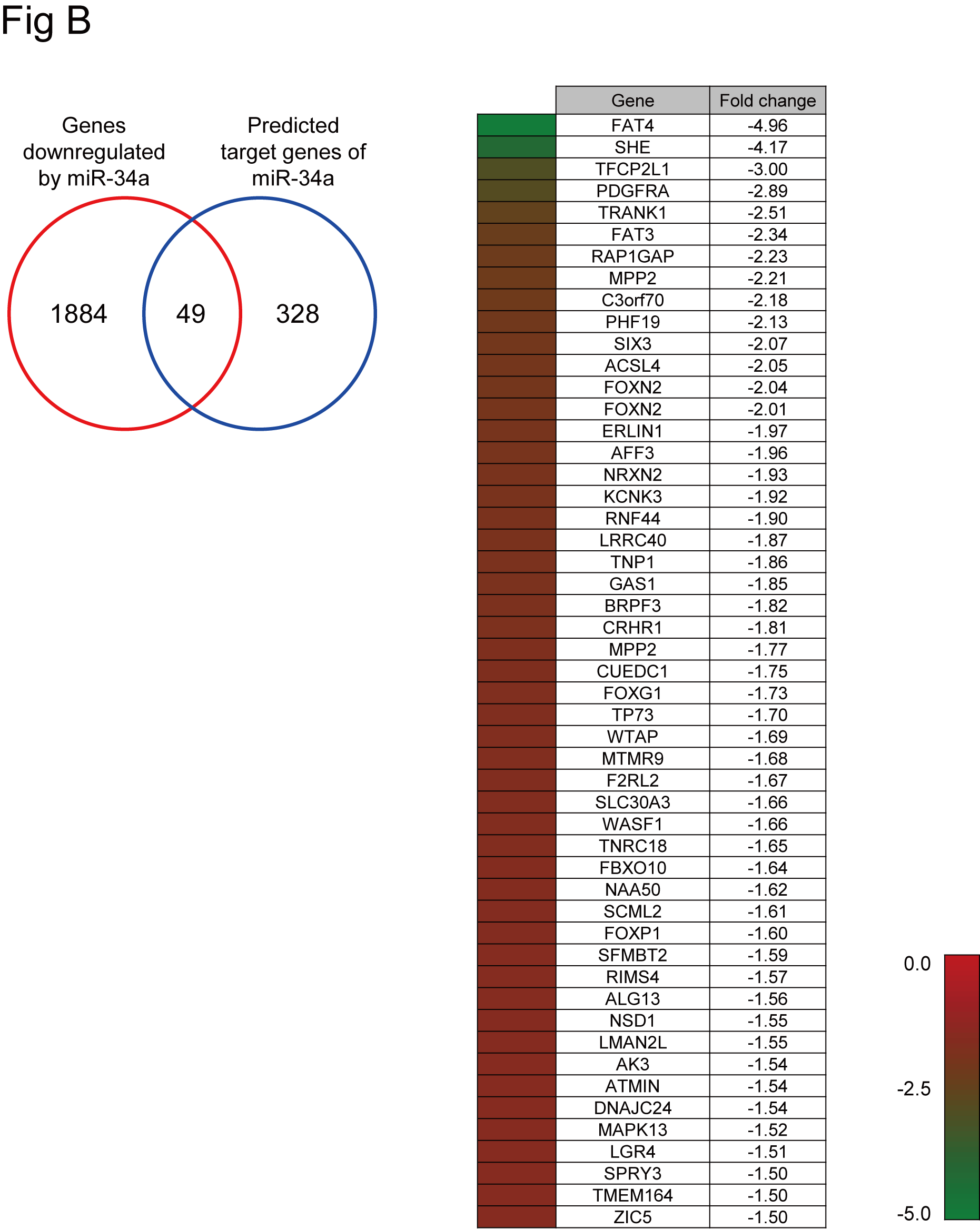


**Fig B**

Venn diagram for genes downregulated by ectopic miR-34a expression in GIST-T1 cells (>1.5-fold) and predicted miR-34a target genes. Downregulated target genes are listed on the right.


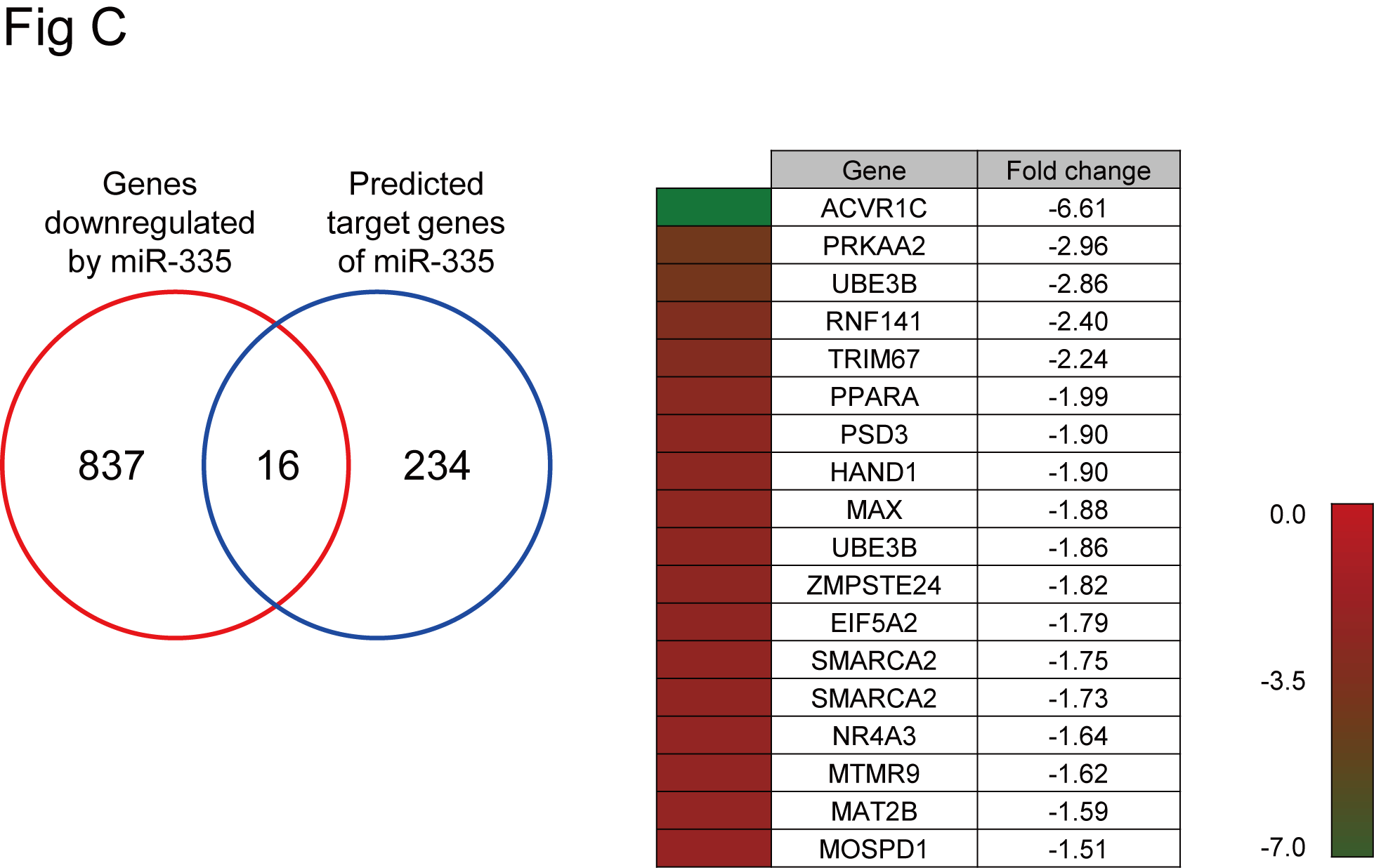


**Fig C**

Venn diagram for genes downregulated by ectopic miR-335 expression in GIST-T1 cells (>1.5-fold) and predicted miR-335 target genes. Downregulated target genes are listed on the right.
